# Comparing Alcohol Policies Between Countries: Science or Silliness?

**DOI:** 10.1371/journal.pmed.0040153

**Published:** 2007-04-24

**Authors:** Alison Ritter

## Abstract

Alison Ritter discusses the pros and cons of a new metric, called the Alcohol Policy Index, which is designed to allow comparisons between countries of their alcohol policies.

It seems self-evident that it would be useful to have a way of comparing alcohol policies between countries. A metric to compare countries would enable improvements or changes to be monitored; create benchmarks for comparative purposes; and potentially improve policy efficiency and effectiveness. Indeed, the World Health Organization (WHO) in its alcohol policy report notes that “…it would be useful to develop a scientific way to measure and to evaluate overall policy comprehensiveness” [1].

But it seems that the endeavour is fraught. The multiplicity of problems—conceptual, methodological, and political—lead some researchers and policy makers to conclude that the effort is not worth pursuing. For those brave or foolhardy enough to persist, including Brand and colleagues who now report their findings in *PLoS Medicine* [2], there are a number of different ways of approaching a common metric. Inputs (such as government spending), outcomes (such as consumption or harm), and policy statements can all be measured and compared. Each of these different approaches has strengths and weaknesses, without a clear front-runner.

## Approaches to a Comparative Index

### Government spending

The “inputs” approach is best characterised by government spending estimates. Measuring government expenditure is one clear way of comparing countries. Indeed, such comparative work has been reported in the literature [3–6]. Interestingly, this work has largely been conducted in the area of illicit drugs, not in the area of alcohol. Drug budgets are a “useful partial description of a nation's drug policy” [5]. The national drug budget can be used to calculate what proportion of a country's gross domestic product is spent on drug policy, enabling country comparisons. And the development of a shared methodology across research teams and countries means that this comparative work is now more sophisticated [6]. However, it has its limitations—for example there is no agreement as to what aspects of government spending should be included [6].

A metric to compare countries would enable improvements or changes to be monitored.

### Cost-of-illness

The greatest criticism of the government spending approach is that it is merely measuring “inputs”, not the consequences of the policies. An alternate approach is that termed cost-of-illness, where the societal cost consequences of drug use (alcohol, tobacco, and/or illicit drugs) are calculated. Again there are a number of methodological stumbling blocks, but much work has been performed internationally to establish agreed-upon methodological standards for the conduct of such studies. In 2003, the WHO published the “International Guidelines for Estimating the Costs of Substance Abuse” [7]. The cost-of-illness approach has its critics. Moore and colleagues have identified a number of improvements that could be made to cost-of-illness studies so that they could be more informative for policy [8].

### Consumption and patterns of use

Consumption rates and patterns of use and associated harms for alcohol and illicit drugs is an obvious way of measuring and comparing countries. The WHO Global Status Report on Alcohol provides data on 189 countries in relation to per capita consumption of alcohol, drinking patterns, and health and social problems associated with alcohol [9]. Countries can be compared using these data—the presumption is that country policies in relation to alcohol drive its associated consumption and harms. It is not clear that one needs to go beyond data such as this to compare countries if one assumes that it is the outcomes of policies that are most important (rather than the existence of policies per se). And in this sense a consumption index represents a more direct metric than the government spending or cost-of-illness approach.

### Burden of disease

Another approach worth mentioning is the burden of disease. The assessment of the extent to which alcohol and other drugs contribute to overall burden of disease has become an important metric as measured by disability-adjusted life years or years of life lost. Clearly the burden of disease has a health focus, but nonetheless represents a single metric for evaluating a country's alcohol responsiveness. The global burden of disease due to alcohol, illicit drugs, and tobacco has been estimated by global region [5]. Once again, there are limitations to this approach (see [5]). The burden of disease approach is more sophisticated than consumption measures alone or cost-of-illness studies, but still lacks an explicit connection with policy responses (although the connection is implied) and includes only the health aspects of alcohol and drug use. It ignores crime effects, social disruption, and economic aspects such as productivity losses.

### Composite harm indices

For illicit drugs, there have been advances in developing composite indices of drug harms capable of providing comparative data either within country across time or between countries.

The 2005 United Nations World Drug Report describes the United Nation's efforts to establish a global Illicit Drug Index for comparing the scale of the problem and monitoring strategic outcomes between countries across the world [10]. The aim is to establish a “single, standard and comparable measure of a country's overall drug problem” [10]. The index measures the extent of the problem (not responses, nor socio-economic impacts). It has three components: production, trafficking, and abuse. The components are combined to produce a single per capita figure for each country.

The Australian Federal Police have been working to produce a drug harm index that represents the dollar value of harm that would have ensued had seized drugs reached the community [11]. The index includes consumption estimates and social cost attributable to particular drugs, and then derives an estimate of an economic cost per kilogram of drug.

In the United Kingdom, the Home Office has developed a drug harm index to monitor changes in drug harm over time [12]. This index combines a number of indicators into one composite measure. Indicators include health consequences (morbidity, mortality), drug-related crime, community perceptions, and drug nuisance (19 harms in total). It incorporates social or economic cost through the weighting of the different harms by economic burden. To be useful for comparisons between countries, the index would need to be calculated for each country. As the authors note, the UK harm index is “purely a measure of realised outcomes”—it does not address the cost-effectiveness of one policy response over another.

### Generalised cost-effectiveness analysis

For an index to include an effectiveness component, it needs to consider the cost-effectiveness of various policy options. The Generalised Cost-Effectiveness Analysis goes some way towards a comparative measure [13]. The approach enables cost-effective interventions to be identified in the context of a country's current consumption and existing interventions. Such an approach is helpful because it provides a sophisticated analysis of the mix of government interventions that are likely to produce the greatest efficiency in terms of costs and benefits. The extent to which this approach can be applied to develop comparisons between countries is unknown. And like all the other measures, it has some significant limitations.

None of the above indices, however, actually measure policy interventions per se. This is why the study in *PLoS Medicine*, which creates a new measure called the Alcohol Policy Index, is unique [2].

## Another Comparative Index—The Alcohol Policy Index

The Alcohol Policy Index is a different metric for comparing countries—it is not cost driven nor does it measure consequences as with the above measures. It could technically be described as an “input” model—i.e., it measures what countries say they do, not what outcomes their policies produce.

As Brand et al. describe, the Alcohol Policy Index rates countries on the extent to which they have implemented alcohol policies. The scores incorporate a weighting that reflects the degree of evidence of effectiveness for each alcohol policy. Having established the scores for a number of countries, the authors then examine the degree of concordance between the country score and the country's consumption levels (as reported in [9]). The authors find a strong relationship between “policy strength” and per capita alcohol consumption. They have approached the exercise with appropriate statistical care.

The index is built around what was known (and published) in 2003 regarding evidence for effectiveness. It excludes research since then on effective alcohol policies, and also excludes those strategies/policies that might work but lack an evidentiary base. The index could be updated with some of the cost-effectiveness work cited in the generalised cost-effectiveness approach [13].

The Alcohol Policy Index has promise, but requires further development. For example, the validity of the index should be examined against a variety of other measures (not just per capita alcohol consumption, but alcohol harms). The major limitation of the Alcohol Policy Index is that it does not value the policy effects, nor link them to any of the above potential measures of policy impact. In addition, as Brand et al. note, it does not accommodate the implementation or enforcement of alcohol policies.

There have been other efforts to develop a scale that measures alcohol policies, and that is capable of being used to compare countries [14,15]. The Alcohol Policy Index and other scales of alcohol control policies provide only one way of enumerating the overall policy stance of a country. A remaining question is: which approach or set of measures will provide the best way forward for comparative analyses?

## Where to From Here?

There are a set of generic problems associated with all of the approaches discussed above, including lack of available data; comparability problems between countries; lack of shared methodology between research groups; and the potential for large differences between regions or states within countries, making country-level comparisons problematic. Country differences include important background variables such as cultural differences; social, political, and economic differences; and epidemiological and demographic differences. These differences may make any index simplified and reductionistic to the point of uselessness. All of the potential indices only include what can be measured, not what would make the most sense conceptually.

If we assume, however, that some of the methodological and conceptual problems can be overcome and that such an index is indeed useful and worthy of scientific pursuit, the question then becomes: which index provides the best possibilities?

I have considered seven different approaches here: government spending is advanced in terms of method and analysis, but provides a limited index; the cost-of-illness and burden of disease approaches calculate the extent of the problem; generalised cost-effectiveness analysis calculates the efficiency of the solutions; and the harm index calculates costs, spending, and harms, but not effectiveness. The Alcohol Policy Index calculates policy implementation. Its flaw is that it measures stated policy objectives and programs, not their implementation nor outcomes (costs, burden, or harms).

Perhaps the challenge from here is to develop a multidimensional index that can accommodate the dimensions of costs, consumption, harms, and cost-effective alcohol control policy responses.

## Abbreviations

WHO: World Health Organization
